# A new stochastic and state space model of human colon cancer incorporating multiple pathways

**DOI:** 10.1186/1745-6150-5-26

**Published:** 2010-04-20

**Authors:** Wai Y Tan, Xiao W Yan

**Affiliations:** 1 Department of Mathematical Sciences, The University of Memphis, Memphis, TN 38152-6429, USA

## Abstract

**Background and Purpose:**

Studies by molecular biologists and geneticists have shown that tumors of human colon cancer are developed from colon stem cells through two mechanisms: The chromosomal instability and the micro-satellite instability. The purpose of this paper is therefore to develop a new stochastic and state space model for carcinogenesis of human colon cancer incorporating these biological mechanisms.

**Results:**

Based on recent biological studies, in this paper we have developed a state space model for human colon cancer. In this state space model, the stochastic system is represented by a stochastic model, involving 2 different pathways-the chromosomal instability pathway and the micro-satellite instability pathway; the observation, cancer incidence data, is represented by a statistical model. Based on this model we have developed a generalized Bayesian approach to estimate the parameters through the posterior modes of the parameters via Gibbs sampling procedures. We have applied this model to fit and analyze the SEER data of human colon cancers from NCI/NIH.

**Conclusions:**

Our results indicate that the model not only provides a logical avenue to incorporate biological information but also fits the data much better than other models including the 4-stage single pathway model. This model not only would provide more insights into human colon cancer but also would provide useful guidance for its prevention and control and for prediction of future cancer cases.

**Reviewers:**

This article was reviewed by M.P. Little and M. Kimmel

## Background

In the past 15 years, molecular biologists and geneticists have revealed the basic molecular and genetic mechanisms for human colon cancer. These mechanisms have been linked to two avenues: The chromosomal instability (CIN) involving chromosomal aberrations and loss of heterozygosity (LOH), and the micro-satellite instability (MSI) involving mis-match repair genes and the creation of mutator phenotype ([1-9]). The pathway of the CIN avenue (also referred to as LOH pathway) involves inactivation through genetic and/or epigenetic mechanisms, or loss, or mutation of the suppressor APC gene in chromosome 5q (about 85% of all human colon cancers) whereas the pathway of the MSI avenue involves mutation or epigenetic inactivation of the mis-match repair suppressor genes (about 15% of all colon cancers). This leads to multiple pathways for the generation of human colon cancer tumors with each pathway following a stochastic multi-stage model and with intermediate transformed cells subjecting to stochastic proliferation (birth) and differentiation (death). The goal of this paper is to develop a stochastic model for human colon cancer to incorporate these biological information and pathways. This paper is an extension of Tan et al. [10], Little and Wright [11] and Little et al. [12]. We note that besides the multiple pathways considered above, Little and Wright [11], Little et al. [12] and Little [13] have also included mixture type of multiple pathways; however, because the mutation rates are very small, the chance of mixture type of pathways will be extremely small in which case the Little model is equivalent to the model in Section 3.

For developing biologically supported stochastic model of carcinogenesis, in Section 2 we present the most recent cancer biology of human colon cancer. Using results from Section 2, we develop in Section 3 a stochastic model for carcinogenesis of human colon cancer involving multiple pathways. In Section 4 we derive a statistical model for cancer incidence data of human colon cancer. By combining models from Sections 3 and 4, in Section 5 we develop a state space model for human colon cancer. In Section 6, by using the state space model in Section 5, we develop a generalized Bayesian inference procedure to estimate unknown parameters and to predict state variables. To illustrate the applications of the model and methods, in Section 7 we apply the model and methods to the colon cancer incidence data from SEER. Finally in Section 8, we discuss the usefulness of the model and methods and provide some conclusions.

### A Brief Summary of Colon Cancer Biology

As discussed in the introduction, genetic studies have indicated that there are two major avenues by means of which human colon cancer is derived: The Chromosomal Instability (CIN) and the Micro-Satellite Instability (MSI). The first avenue is associated with the LOH pathway involving the APC gene in chromosome 5q and the latter associated with the micro-satellite pathway involving mis-match repair genes. The most important oncogene is the *β*-catenin gene in chromosome 3p22.

### The CIN (LOH) Pathway of Human Colon Cancer (The APC-*β-catenin - Tcf - myc *pathway)

The CIN pathway involves loss or inactivation of the tumor suppressor genes - the APC gene in chromosome 5q, the Smad-4 gene in chromosome 18q and the p53 gene in chromosome 17p; see **Remark 1**. This pathway accounts for about 85% of all colon cancers. It has been referred to as the LOH pathway because it is characterized by aneuploidy/or loss of chromosome segments (chromosomal instability); see **Remark 2**. This pathway has also been referred to as APC-*β *- *catenin - Tcf - myc *pathway because it involves the destruction complex GSK-3*β *-Axin-APC which phosphorylates the *β*-catenin protein leading to its degradation; when both copies of the APC gene are inactivated or mutated, the destruction complex is then inactive leading to accumulation of free *β*-catenin proteins in the cytoplasm which move to the nucleus to complex with Tcf/Lef transcription factor to activate and transcript oncogenes myc, cyclin D and CD44. (Free *β*-catenin protein in the cytoplasm also binds with E-cadherin and *α*-catenin to disrupt the gap junction between cells, leading to migration and metastasis of cancer tumors.)

**Figure 1:**
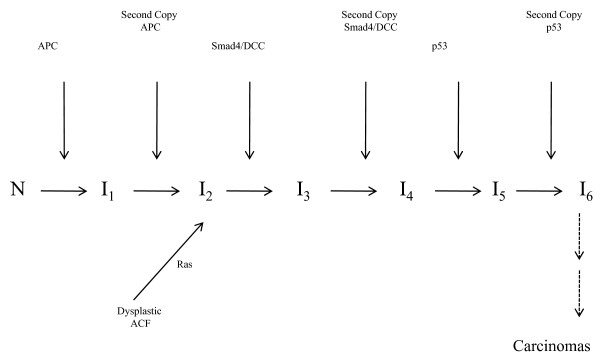
**The CIN pathway of human colon cancer**. Sporatic Chromsomal Instability Pathways of human colon cancer.

Morphological studies have indicated that inactivation, or loss or mutation of APC creates dysplastic aberrant crypt foci (ACF) which grow into dysplastic adenomas. These adenomas grow to a maximum size of about 10 mm^3^; further growth and malignancy require the abrogation of differentiation, cell cycle inhibition and apoptosis which are facilitated by the inactivation, or mutation or loss of Smad-4 gene in 18q and the p53 gene in 17p. The mutation or activation of the oncogene H-ras in chromosome 11p and/or mutation and/or activation of the oncogene src in chromosome 20q would speed up these transitions by promoting the proliferation rates of the respective intermediate initiated cells [14]. This pathway is represented schematically by Figure 1.

The model in Figure 1 is a 6-stage model. However, because of the haplo-insufficiency of the Smad4 gene (see Alberici et al.[15]) and the haplo-insufficiency of the p53 gene ([16]), one may reduce this 6-stage model into a 4-stage model by combining the third stage and the fourth stage into one stage and by combining the fifth stage and the sixth stage into one stage. This may help explain why for single pathway models, the 4-stage model fits the human colon cancer better than other single pathway multi-stage models ([17]). Recent biological studies by Green and Kaplan [4] and others have also shown that the inactivation or deletion or mutation of one copy of the APC gene in chromosome 5 can cause defects in microtubule plus-end attachment during mitosis dominantly, leading to aneuploidy and chromosome instability. This would speed up the mutation or inactivation of the second copy of the APC gene and increase fitness of the APC-carrying cells in the micro-evolution process of cancer progression. This could also help explain why the APC LOH pathway is more frequent than other pathways.

**Remark 1: **As observed by Sparks et al. [8], instead of the APC gene, this pathway can also be initiated by mutation of the oncogene *β*-catenin gene; however, the proportion of human colon cancer due to mutation of *β*-catenin is very small (less than 1%) as compared to the APC gene, due presumably to the contribution of the APC on chromosome instability ([4]). Similarly, the destruction complex can become inactive either by the inhibition of GSK-3*β *through the Wnt signalling pathway (see [18]) or the inactivation or mutation of the Axin protein, leading to accumulation of the *β*- Catenin proteins in the cytoplasm; but the proportion of colon cancer caused by inhibition of GSK-3*β *is also very small as compared to the colon cancer cases caused by the CIN and the MSI pathways.

**Remark 2: **The APC gene in chromosome 5q acts both as a tumor suppressor gene and an oncogene in initiating and promoting colon carcinogenesis. As an oncogene, the APC gene acts dominantly in regulating microtubule plus-end attachment during mitosis ([4]). Thus, the inactivation or deletion or mutation of one copy of the APC gene in chromosome 5 can cause defects in microtubule plus-end attachment during mitosis, leading to aneuploidy and chromosome instability. This would speed up the mutation or inactivation of the second copy of the APC gene and increase fitness of the APC-carrying cells in the micro-evolution process of cancer progression. This could also help explain why the APC LOH pathway is more frequent than other pathways.

### The MSI (Micro-Satellite Instability) Pathway of Human Colon Cancer

**Figure 2:**
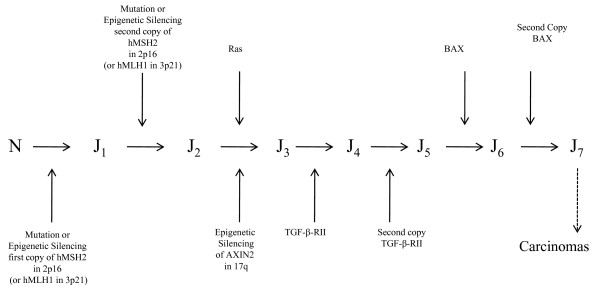
**The MSI pathway of human colon cancer**. Microsatellite Instability pathway of human colon cancer.

This pathway accounts for about 15% of all colon cancers and appears mostly in the right colon. It has been referred to as the MSI pathway or the mutator phenotype pathway because it is initiated by the mutations or epigenetic methylation of the mis-match repair genes (mostly hMLH1 in chromosome 3p21 and hMSH2 in chromosome 2p16) creating a mutator phenotype to significantly increase the mutation rate of many critical genes 10 to 1000 times. Normally these critical genes are TGF-*β RII*, Bax (The X protein of bcl-2 gene), IGF2R, or CDX-2. The mis-match repair genes are hMLH1, hMSH2, hPMS1, hPMS2, hMSH6 and hMSH3; mostly hMLH1 (50%) and hMSH2 (40%). This pathway is represented schematically by Figure 2. As in the LOH pathway, assuming haplo-insufficiency of tumor suppressor genes, one may approximate this pathway by a 5-stage model.

Morphologically, mutation or methylation silencing of the MMR gene hMLH1 or hMSH2 generates hyperplastic polyps which lead to the generation of serrated adenomas. These adenomas develop into carcinomas after the inactivation, or loss or mutations of the TGF-*β RII *gene and the Bax gene, thus abrogating differentiation and apoptosis. (Bax is an anti-apoptosis gene.) In what follows, we let N denote the normal stem cells, *J*_*i *_the *i-*th stage cells in the MSI pathways. Then for sporadic MSI, the model is *N *→ *J*_1 _→ *J*_2 _→ *J*_3 _→ *J*_4 _→ *J*_5 _→ *cancer tumor*.

### The Major Signalling Pathways for Human Colon Cancer

Recent biological studies ([18,19]) have shown that both the CIN and the MSI pathways involve the Wnt signalling pathway and the destruction complex (this complex is a downstream of the Wnt signalling pathway), the TGF-*β *inhibiting signalling pathway and the p53-Bax apoptosis signalling pathway, but different genes in the CIN and MSI pathways are affected in these signalling processes. In the CIN pathway, the affected gene is the APC gene in the Wnt signalling, the Smad4 in the TGF-*β *signalling and the p53 gene in the p53-Bax signalling; on the other hand, in the MSI pathway, the affected gene is the Axin 2 gene in the Wnt signalling, the TGF-*β *-Receptor II in the TGF-*β *signalling and the Bax gene in the p53-Bax signalling.

Because the probability of point mutation or genetic changes of genes are in general very small compared to epigenetic changes, one may speculate that colon cancer may actually be initiated by some epigenetic mechanisms ([18,20,21]). In fact, Breivik and Gaudernack [20] showed that in human colon cancer, either methylating carcinogens or hyper-methylation at *C*_*p*_*G *islands would lead to G/T mismatch which in turn leads to Mis-match Repair (MMR) gene deficiency or epigenetic silencing of the MMR genes and hence MSI (Micro-satellite Instability); alternatively, either hypo-methylation, or bulky-adduct forming (BAF) carcinogens such as alkylating agents, UV radiation and oxygen species promote chromosomal rearrangement via activation of mitotic check points (MCP), thus promoting CIN (Chromosomal Instability). A recent review by Baylin and Ohm [18] have demonstrated that epigenetic events may lead to LOH and mutations of many genes which may further underline the importance of epigenetic mechanisms in cancer initiation and progression.

Based on the above biological studies, in this paper we thus postulate that the incidence data of human colon cancer are described and generated by a multi-stage model involving 2 pathways as defined above. In this paper, because of haploid-insufficiency of the tumor suppressor genes {*Smad*_4_, *p*53, *Axin, Bax, TGF - β - ReceptorII*}, the number of stages for the CIN pathway and MSI are assumed as 4 and 5 respectively.

## Methods

### Stochastic Multi-Stage Model of Carcinogenesis for Human Colon Cancer Involving Multiple Pathways

**Figure 3:**
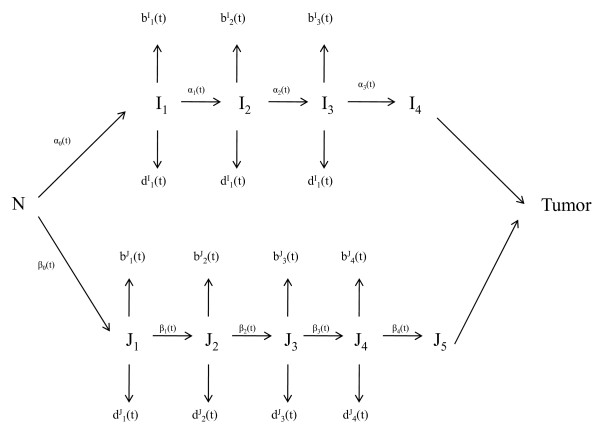
**The multiple pathways of human colon cancer**. All pathways involoved in sporatic human colon cancer.

From results of Section 2, it follows that the stochastic multi-stage model for human colon cancer can be represented schematically by Figure 3.

In Figure 3, the model assumes that cancer tumors are generated by two pathways with pathway 1 as a *k*_1_-stage multi-stage model involving *I*_*l *_(*l *= 1, ..., *k*_1_) cells and with pathway 2 as a *k*_2_-stage multi-stage model involving *J*_*r *_(*r *= 1, ..., *k*_2_) cells. (For human colon cancer, *k*_1 _= 4, *k*_2 _= 5.) The state variables are then  (*t*) = {*I*_*l*_(*t*), *l *= 1, ..., *k*_1 _- 1, *J*_*r*_(*t*), *r *= 1, ..., *k*_2 _- 1} and *T*(*t*), where *T*(*t*) denotes the number of cancer tumors at time *t *and where *I*_*l*_(*t*) (*J*_*r*_(*t*)) denote the number of the *I*_*l *_(*J*_*r*_) initiated cells for {*l *= 1, ..., *k*_1 _- 1 (*r *= 1, ..., *k*_2 _- 1)} respectively. Notice that because cell proliferation, cell differentiation and apoptosis, mutation or genetic changes all occur during cell division and cell division cycle, and because  (*t *+ Δ*t*) develop from  (*t*) through cell divisions during (*t*, *t *+ Δ*t*], one may pratically assume that ( (*t*), *t *≥ * t*_0_) is a Markov process with continous time, where *t*_0 _represents time at birth; one the other hand, *T*(*t *+ Δ*t*) may derive from  () cells before time t, *T*(*t*) is in general not Markov ([22,23])). If one assumes that the  and  cells grow instantaneously into cancer tumors as soon as they are generated, then one may also assume the *T*(*t*) as Markov. In this case, as illustrated in Tan[24], one may use standard Markov theory to derive the probability generating function (pgf) of the probabilities of these variables and hence the probability distribution of these variables. Let *ψ *(*x*_*l*_, *l *= 1,..., *k*_1 _-1, *y*_*r*_, *r *= 1,..., *k*_2_-1, *z*; *t*_0_, *t*) = *ψ *(, , *z*; *t*_0_, *t*) denote the pgf of { (*t*), *T*(*t*)}. Let  denote the mutation rates, the birth rates and the death rates of {*I*_*l*_, *J*_*r*_} cells as given in Table 1 respectively.

**Table 1:** Transition rates and transition probabilities for human colon carcinogenesis

1 N	→	1 N, 1 *I*_1_	*α*_0_(*t*)Δ*t*
1 N	→	1 N, 1 *J*_1_	*β*_0_(*t*)Δ*t*
1 *I*_*l*_	→	2 *I*_*l*_	
1 *I*_*l*_	→	death	
1 *I*_*l*_	→	1 *I*_*l*_,1 *I*_*l*+1_	*a*_*l*_(*t*)Δ*t*
	*l *= 1, ..., *k*_1 _- 1		
1 *J*_*r*_	→	2 *J*_*r*_	
1 *J*_*r*_	→	death	
1 *J*_*r*_	→	1 *J*_*r*_,1 *J*_*r*+1_	*β*_*r*_(*t*)Δ*t*
	*r *= 1, ..., *k*_2 _- 1		

If *T*(*t*) is Markov, then by using the method of Kolmogorov forward equation of these variables (Tan [24]), it can readily be shown that *ψ *(, , *z*; *t*_0_, *t*) satisfies the following partial differential equation (pde):(1)

where *λ*_*I *_(*t*) = *N*(*t*)*α*_0_(*t*), *λ*_*J *_(*t*) = *N*(*t*) *β*_0_(*t*), , , and the initial condition is *ψ *(, , *z*; *t*_0_, *t*_0_) = 1 given normal individuals at risk at time *t*_0 _.

The above pde is in general very difficult to solve; further, even if the solution of this equation can be derived, the results are very difficult to apply to estimate the unknown parameters and to predict future cancer cases. Most importantly, *T*(*t*) may not be Markov so that this theory is not applicable (Fakir et al.[22,23]). In this paper, we will thus propose an alternative approach through stochastic equations. It can easily be shown through the method of pgf that if *T*(*t*) is Markov, then the stochastic equation method is equivalent to the method of Markov theory; as we shall see, however, the stochastic equation method is more powerful and does not need to assume Markov for *T*(*t*).

### The Stochastic Equation for State Variables

To derive stochastic equations for the state variables, let  be the number of births of the *I*_*l *_(*J*_*r*_) initiated cells during (*t*, *t *+ Δ*t*] {*l *= 1, ..., *k*_1_- 1 (*r *= 1, ..., *k*_2 _- 1)},  the number of deaths of the *I*_*l *_(*J*_*r*_) initiated cells during (*t*, *t *+ Δ*t*] {*l *= 1, ..., *k*_1_- 1 (*r *= 1, ..., *k*_2 _- 1)} and  the number of mutation (*I*_*l *_→ *I*_*l*+1_) (*J*_*r *_→ *J*_*r*+1_) of *I*_*l *_(*J*_*r*_) cells during (*t*, *t *+ Δ*t*] {*l *= 1, ..., *k*_1 _- 1 (*r *= 1, ..., *k*_2 _- 1)}.

Also let  be the number of mutation of *N *→ *I*_1_(*N *→ *J*_1_) during (*t*, *t *+ Δ*t*]. Taking into account of all possible input and output of relevant cells, we have the following stochastic equations for the state variables:(2)

Because the transition variables  are random variables, the above equations are stochastic equations. With the transition rates as given in Table 1, it can readily be shown that to the order of *o*(Δ*t*), the conditional probability distributions of  and  given *N*(*t*) are Poisson with means *λ*_*I*_(*t*)Δ*t *and *λ*_*I*_(*t*)Δ*t *respectively whereas the conditional probability distributions of the numbers of births and deaths given the staging variables (i.e. the *I*_*l*_(*t*) and *J*_*r*_(*t*)) follow multinomial distributions independently. That is,(4)

for *l *= 1, 2, ..., *k*_1 _- 1,(6)

for *r *= 1, ..., *k*_2 _- 1,(7)

where *λ*_*I*_(*t*) = *N*(*t*)*α*_0_(*t*), *λ*_*J *_(*t*) = *N*(*t*)*β*_0_(*t*).

Because the number of mutations of the *I*_*l *_cells would not affect the size of the *I*_*l *_population but only increase the number of *I*_*l*+1 _cells and because the mutation rate of *I*_*l *_cells is very small (10^-5 ^~10^-8^), it can readily be shown that to the order of *o*(Δ*t*), the conditional probability distribution of  given *I*_*l*_(*t*) *I*_*l *_cells at time *t *is Poisson with mean *I*_*l*_(*t*)*α*_*l*_(*t*)Δ*t *independently of  and other transition variables. That is,(8)

independently of  and other transition variables.

Similarly, we have that to the order of *o*(Δ*t*),(9)

independently of  and other transition variables.

Using the probability distributions given by equations (5)-(10) and by subtracting from the transition variables the conditional expected values respectively, we have the following stochastic differential equations for the staging state variables:(10)

where .

In the above equations, the random noises  are derived by subtracting the conditional expected numbers from the random transition variables respectively. Obviously, these random noises are linear combinations of Poisson and multinomial random variables. These random noises have expected value zero and are un-correlated with the state variables {*I*_*l*_(*t*), *l *= 1, ..., *k*_1 _- 1, *J*_*r*_(*t*), *r *= 1, ..., *k*_2 _- 1}. It can also be shown that to the order of *o*(Δ*t*), these random noises are uncorrelated with one another and have variances given by:

where *I*_0_(*t*) = *J*_0_(*t*) = *N*(*t*).

### The Expected Numbers

Let *u*_*I*_(*l*, *t*) = *E *[*I*_*l*_(*t*)] and *u*_*J *_(*r*, *t*) = *E *[*J*_*r*_(*t*)] denote the expected numbers of *I*_*l*_(*t*) and *J*_*r*_(*t*) respectively and write *u*_*I*_(0, *t*) = *u*_*J*_(0, *t*) = *N*(*t*). Using equations (11)-(12), we have the following differential equations for these expected numbers:(12)

The solution of the above equations are:

If the model is time homogeneous, then *λ*_*I*_(*t*) = *λ*_*I*_, *λ*_*J*_(*t*) = *λ*_*J*_,  for *l *= 1, ..., *k*_1 _- 1 and  for *r *= 1, ..., *k*_2 _- 1. If the proliferation rates are not zero and if  for all *l *≠ * u *and *r *≠ * v*, then the above solutions reduce to:

where .

### The Probability Distribution of State Variables and Transition Variables

Although *T*(*t*) is not Markov, the random vector { (*t*), *t *≥ * t*_0_} is Markov with continuous time. To derive the transition probability of this process, denote by *f*(*x*, *y *: *N*, *p*_1_, *p*_2_) the density at (*x*, *y*) of the multinomial distribution *ML*(*N*; *p*_1_, *p*_2_) with parameters (*N*; *p*_1_, *p*_2_) and *h*(*x*; *λ*) the density at *x *of the Poisson distribution with mean *λ*. Then, using the probability distributions given by equations (5)-(10), the transition probability of this Markov process is, to order of *o*(Δ*t*):

where *I*_0_(*t*) = *J*_0_(*t*) = *N*(*t*), *a*(*l*_*u*_, *i*_*u*_; *t*) = *I*_*u*_(*t *+ Δ*t*) - *I*_*u*_(*t*) - *l*_*u *_+ *i*_*u*_, *u *= 1, ..., *k*_1 _- 1 and where *b*(*m*_*v*_, *j*_*v*_; *t*) = *J*_*v*_(*t *+ Δ*t*) - *J*_*v*_(*t*) - *m*_*v *_+ *j*_*v*_, *v *= 1, ..., *k*_2 _- 1.

The above transition probability and hence the probability distribution of  (*t*) is too complicated to be of much use. For implementing the Gibbs sampling procedure to estimate parameters and to predict state variables, we use data augmentation method to expand the model. Thus, we define the augmented variables . (In what follows we will refer these variables as the transition variables, unless otherwise stated.)

Put . Then { (*t*), *t *≥ *t*_0_} is Markov with continuous time. Using the probability distributions of the transition random variables given by equations (5)-(10), the transition probability *P*{ (*t *+ Δ*t*)| (*t*)} is(14)

where(15)

and(16)

where  for *l *= 1, ..., *k*_1 _- 1 and  for *r *= 1, ..., *k*_2 _- 1.

The probability distribution given by equation (15) will be used to derive estimates and predicted numbers of state variables. This is discussed in Section 6.

### A Statistical Model and The Probability Distribution of the Number of Detectable Tumors

The data available for modeling carcinogenesis are usually cancer incidence over different time periods. For example, the SEER data of NCI/NIH for human cancers are given by {(*y*_*j*_, *n*_*j*_), *j *= 1, ..., *n*}, where *y*_*j *_is the observed number of cancer cases during the *j-*th age group and *n*_*j *_is the number of normal people who are at risk for cancer and from whom *y*_*j *_of them have developed cancer during the age group. Given in Table 2 are the SEER data for human colon cancer adjusted for genetic cancer cases.

**Table 2:** Colon Cancer Data from SEER(overall population)

Age Group	Number of People at Risk	Observed Colon Cancer Cases	Total Prediced Colon Cancer
0	9934747	1	0
0-4	38690768	2	0
5-9	48506058	2	6
10-14	49881935	35	44
15-19	50447512	104	164
20-24	51612785	337	370
25-29	54071811	847	965
30-34	54194486	1829	2080
35-39	50363957	3420	3534
40-44	46029771	6174	6698
45-49	40674188	10950	11072
50-54	36070434	18716	18256
55-59	31084543	27438	25875
60-64	26507762	37155	34867
65-69	22772688	47202	45156
70-74	18785224	53190	52810
75-79	14592602	52887	53479
80-84	9751212	42589	41517

The predicted numbers were generated by the model with unknown paprameters being substituted by the estimates respectively.

### The Probability Distribution of the Number of Detectable Tumors for Colon Cancer

To derive the probability distribution of time to tumors, one needs the probability distribution of *T*(*t*). For deriving this probability distribution, we observe that malignant cancer tumors arise by clonal expansion from primary  cells and primary  cells, where primary  cells are  cells derived from  cells by mutation of  cells and primary  cells are  cells derived from  cells by mutation of  cells.

Let  be the probability that a primary  () cancer cell at time s develops into a detectable cancer tumor at time t. Let *T*_*i*_(*t*) be the number of cancer tumors derived from the *i-*th pathway. Then, to order of *o*(Δ*t*), the conditional probability distribution of *T*_1_(*t*) given { (*s*), *s *≤ *t*} is Poisson with mean *ω*_1_(*t*) independently of *T*_2_(*t*), where

Similarly, to order of *o*(Δ*t*), the conditional probability distribution of *T*_2_(*t*) given { (*s*), *s *≤ *t*} is Poisson with mean *ω*_2_(*t*) independently of *T*_1_(*t*), where

Let *Q*_*i*_(*j*) (*i *= 1, 2) be defined by:

where *R*_*i*_(*t*_*j*-1_, *t*_*j*_) = *ω*_*i*_(*t*_*j*-1_) - *ω*_*i*_(*t*_*j*_).

Then *Q*_*i*_(*j*) is the probability that cancer tumors would develop during the *j-*th age group by the *i-*th pathway. Since cancer tumors develop if and only if at least one of the two pathways yield cancer tumors, the probability that each normal person at time *t*_0 _will develop cancer tumors during (*t*_*j*-1_, *t*_*j*_] is given by *Q*_*T *_(*j*), where

For practical applications, we observe that to order of *o*( (*t*)) and *o*( (*t*)) respectively, the *ω*_*i*_(*t*) in *Q*_*i*_(*j*) are approximated by

Similarly, it can readily be shown that to the order of *Min*{*o*( (*t*)), *o*( (*t*)}, *Q*_*T*_(*t*) ~*Q*_1_(*t*) + *Q*_2_(*t*).

To further simplify the calculation of *Q*_*T *_(*j*), we observe that in studying human cancers, one time unit (i.e. Δ*t *= 1) is usually assumed to be 3 months or 6 months or longer. In these cases, one may practically assume  and  if *t *- *s *≥ 1.

### A Statistical Model for Cancer Incidence Data

Let *y*_*j *_be the observed number of the number of cancer cases *Y*_*j *_developed during (*t*_*j*-1_, *t*_*j*_] given *n*_*j *_people at risk for cancer, who are normal at birth (*t*_0_). We assume that each individual develops colon cancer tumor by the same mechanism independently of one another. Then for each person who is normal at birth (*t*_0_), the probability that this individual would develop colon cancer tumor during the *j*-th age group (*t*_*j*-1_, *t*_*j*_] is given by *Q*_*T *_(*j*). It follows that the probability distribution of *Y*_*j *_given that *n*_*j *_is:(17)

Because *n*_*j *_is very large and *Q*_*T *_(*j*) is very small, approximately *Y*_*j *_is Possion with mean *τ*_*j *_= *n*_*j*_*Q*_*T *_(*j*). Notice that to the order of *Max*{*o*( (*t*)), *o*( (*t*))}, *τ*_*j *_(and hence the probability distribution of *Y*_*j*_) depends on the stochastic model of colon carcinogenesis through the expected number {*E *[ (*t*)], *E *[ (*t*)]} of { (*t*),  (*t*)} and the parameters { (*t*),  (*t*)} over the time period (*t*_*j*-1_, *t*_*j*_].

### The State Space Model of Human Colon Cancer

State space model is a stochastic model which consists of two sub-models: The stochastic system model which is the stochastic model of the system and the observation model which is a statistical model based on available observed data from the system. Thus, the state space model of a system takes into account the basic mechanisms of the system and the random variation of the system through its stochastic system model and incorporates all these into the observed data from the system; furthermore, it validates and upgrades the stochastic model through its observation model and the observed data of the system. As illustrated in Tan ([25], Chapters 8-9), the state space model has many advantages over both the stochastic model and the statistical model when used alone since it combines information and advantages from both of these models.

For human colon cancer, the stochastic system model of the state space model is the stochastic model consisting of 2 pathways with each pathway following a multi-stage model as described in Section 3; the observation model of this state space model is a statistical model based on the observed number of colon cancer cases as described in Section 4.

### The Stochastic System Model and the State Variables

Putting Δ*t *= 1 for some fixed small interval, then the staging variables are ***X ***= { (*t*), *t *= *t*_0_, *t*_0 _+ 1, ..., *t*_*M*_} and the transition variables are ***U ***= { (*t*), *t *= *t*_0_, *t*_0 _+ 1, ..., *t*_*M *_- 1}. From results in Section (3.3), the joint probability distribution of {***X***, ***U***} given the parameters Θ is:(18)

where *P*{ (*t - *1)| (*t - *1)} and *P*{ (*t*)| (*t - *1),  (*t - *1)} are given by equations (16) and (17) respectively and where Θ = {*λ*_*I*_, *λ*_*J*_, *α*_*l*_(*t*), *β*_*r*_(*y*), , *d*_*l*_(*t*)^(*I*)^(*t*), , *d*_*r*_(*t*)^(*J*)^(*t*), *l *= 1, ..., *k*_1 _- 1, *r *= 1, ..., *k*_2 _- 1}.

Notice that this probability distribution is basically a product of Poisson distributions and multinomial distributions.

### The Observation Model Using SEER Data

Put ***Y ***= (*Y*_*j*_, *j *= 1, ..., *m*) and  = (*y*_*j*_, *j *= 1, ..., *m*)'. By the probability distribution given by equation (18), the conditional probability density of ***Y ***given {***X***, ***U***, Θ} is approximately given by:(19)

where *h*(*Y*_*j*_; *τ*_*j*_) is the density at *Y*_*j *_of the Poisson distribution with mean *τ*_*j*_.

Then the likelihood function of Θ given (***X***, ***U***) is . It follows that the deviance from this density is:(20)

where  and  is the maximum likelihood estimate of *τ*_*j*_.

From equations (19)-(20), we have for the joint density of (***X***, ***U***, ***Y ***) given Θ:(21)

To apply the above distribution to estimate unknown parameters and to fit real data, we also make the following assumptions: (a) From biological observations ([1-9]), one may practically assume that {*α*_*l*_(*t*) = *α*_*l*_, *l *= 0, 1, 2, 3; *β*_*r *_(*t*) = *β*_*r*_, *r *= 0, 1, 2, 3, 4, }. (b) Because the colon polyps are generated by proliferation of *I*_2 _cells and *J*_3 _cells and because the polyps can only grow to a maximum size of about 10 *mm*^3^, we assume that  and  for some small (*δ*_*i *_> 0, *i *= 1, 2). (c) Because colon cell divisions are mainly due to action of the *β*-catenin gene, one may also assume . In this case, one has approximately  and , *r *= 1, 2. Under these assumptions, the unknown parameters of interest are Θ = {Θ_1_, Θ_2_}, where  and Θ_2 _= {*α*_3_, *β*_4_).

### The Generalized Bayesian Method and the Gibbs Sampling Procedure

The generalized Bayesian inference is based on the posterior distribution *P*{Θ|***X***, ***U***, } of Θ given {***X***, ***U***, ***Y ***= }. This posterior distribution is derived by combining the prior distribution *P*{Θ} of Θ with the probability distribution *P*{***X***, ***U***, ***Y***|Θ} given by equation (20) with ***Y ***being replaced by . It follows that this inference procedure would combine information from three sources: (1) Previous information and experiences about the parameters in terms of the prior distribution *P*{Θ}, (2) Biological information represented by the stochastic system equations of the stochastic system (*P*{***X***, ***U***|Θ}) and (3) Information from observed data, represented by the statistical model through the conditional likelihood *L*(Θ|, ***X***, ***U***).

Because of additional information from the stochastic system model, this inference procedure is advantageous over the standard Bayesian procedure in that it can avoid the identifiability problems associated with standard Bayesian method. For example, we have shown that to the order of *Max*{*o*(*α*_3_(*t*)), *o*(*β*_4_(*t*))} the probability distribution of the *Y*_*j*_'s depends on the stochastic model through the expected numbers of *I*_3_(*t*) and *J*_4_(*t*), which depend on the birth rates and death rates only through the difference of these rates. It follows that it is not possible to estimate the birth rates and death rates separately by the traditional Bayesian method. Most importantly, the number of parameters is very large and the number of data points is limited. Thus, without information from the stochastic system model, it is virtually impossible to estimate all unknown parameters; for more examples, see Tan ([25,26]).

### The Prior Distribution of the Parameters

For the prior distributions of Θ, because biological information have suggested some lower bounds and upper bounds for the mutation rates and for the proliferation rates, we assume(22)

where c is a positive constant if these parameters satisfy some biologically specified constraints; and equal to zero otherwise. These biological constraints are:

(i) For the mutation rates of the *I*_*i *_cells in the LOH pathway, 1 <*λ*_*I *_< 1000 (*N *→ *I*_1_), 10^-6 ^*<α*_*i *_< 10^-4^, *i *= 1, 2, 3. For the proliferation rates of *I*_*i*_cells in the LOH pathway, *γ*_1_(*t*) = 0, 0 < < 0.5, *i *= 2, 3, *γ*_2_(*t*) = , 10^-4 ^<*γ*_2 _< 2 * 10^-2^, 10^-5 ^<*δ*_1 _< 5 * 10^-3^, 10^-2 ^<*γ*_3_< 0.5.

(ii) For the mutation rates in the MSI pathway, 1 <*λ*_*J*_< 1000 (*N *→ *I*_1_), 10^-8 ^<*β*_1 _< 10^-5^, 10^-6 ^<*β*_*j *_< 10^-2^, *j *= 2, 3, 4. For the proliferation rates in the MSI pathway, .

We will refer the above prior as a partially informative prior which may be considered as an extension of the traditional non- informative prior given in Box and Tiao [27].

**The Posterior Distribution of the Parameters Given **{***Y ***= , ***X***, ***U***}

Combining the prior distribution given in (6.1) with the density of *P*{***X***, ***U***, ***Y ***|Θ} given in equation (20), one can readily derive the conditional posterior distribution of Θ given {***X***, ***U***, ***Y ***= }. For (*l *= 2, 3), denote by:  and ; similarly, for *r *= 3, 4, we define {*B*_*rJ*_, *D*_*rJ*_, *N*_*rJ*_} by replacing  by  respectively. Then, we have the following results for the conditional posterior distributions:

(i) The conditional posterior distributions of Θ_1_(1) = {*λ*_*I*_, *λ*_*J*_, *α*_*l*_, *l *= 1, 2, *β*_*r*_, *r *= 1, 2, 3} given {***X***, ***U***, ***Y ***= } is:

(ii) The conditional posterior distributions of  given {***X***, ***U***, ***Y ***= } is:

(iii) The conditional posterior distribution of {*α*_3_, *β*_4_} given {***X***, ***U***, ***Y ***= } is:

(vi) The conditional posterior distribution of  given {***X***, ***U***, ***Y ***= } and the conditional posterior distribution of  given {***X***, ***U***, ***Y ***= } are represented respectively by:

### The Multi-level Gibbs Sampling Procedure For Estimating Parameters

Given the above probability distributions, the multi-level Gibbs sampling procedure for deriving estimates of the unknown parameters are given by:

(a) Step 1: Generating (***X***, ***U***) Given (***Y ***= , Θ) (The Data-Augmentation Step):

Given ***Y ***=  and given Θ, use the stochastic equations (3)-(4) and the probability distributions given by equations (5)-(10) in Section 3 to generate a large sample of (***X***, ***U***). Then, by combining this sample with *P*{***Y ***= |***X***, ***U***, Θ} to select (***X***, ***U***) through the weighted bootstrap method due to Smith and Gelfant [28]. This selected (***X***, ***U***) is then a sample from *P*{***X***, ***U***|***Y ***= , Θ} even though the latter is unknown. (For proof, see Tan [25], Chapter 3.) Call the generated sample (, ).

(b) Step 2: Estimation of Θ = {Θ_1_, Θ_2_} Given {***Y ***= , ***X***, ***U***}:

Given ***Y ***=  and given (***X***, ***U***) = (, ) from Step 1, derive the posterior mode of the parameters by maximizing the conditional posterior distribution *P*{Θ|, , }. Denote the generated mode as .

(c) Step 3: Recycling Step.

With {(***X***, ***U***) = (, ), Θ = } given above, go back to Step (a) and continue until convergence. The convergence of the above steps can be proved using procedure given in Tan ([25], Chapter 3). At convergence, the  are the generated values from the posterior distribution of Θ given ***Y ***=  independently of (***X***, ***U***) (for proof, see Tan [25], Chapter 3). Repeat the above procedures one then generates a random sample of Θ from the posterior distribution of Θ given ***Y ***= ; then one uses the sample mean as the estimates of (Θ) and use the sample variances and covarainces as estimates of the variances and covariances of these estimates.

## Results

### Application to Fit the SEER Data

In this section, we will apply the above model to the NCI/NIH colon cancer data from the SEER project. Given in Table 2 are the numbers of people at risk and colon cancer cases in the age groups together with the predicted cases from the model. There are 18 age groups with each group spanning over 5 years. To fit the data, we have assumed that  for *j *= 1, 2 because of the observation that uncontrolled cell division of colon stem cells is mainly initiated by the oncogene *β*-catenin in 3p22. Given in Table 3 are the estimates of the mutation rates, the birth rates and the death rates of the *I*_*i *_cells and *J*_*j*_cells. Given in Figure 3 is the plot of probability density of time to tumors.

**Table 3:** Estimates of Parameters for Each Pathway

	LOH Pathway				
	***I*_0_**	***I*_1_**	***I*_2_**	***I*_3_**	
Mutation Rate	1.4E-06	2.2E-04	3.2E-03	1.2E-06	
	± 1.69E-08	± 1.32E-05	± 3.33E-04	± 2.06E-07	
Proliferation Rate	0	0	3.6E-03	1.6E-02	
	N/A	N/A	± 1.12E-03	± 4.78E-04	
Birth Rate Para.	0	0	7.4E-03	1.9E-02	
	N/A	N/A	± 1.03E-03	± 4.08E-04	
Growth Limiting Para.	N/A	N/A	8.3E-05	N/A	
	N/A	N/A	± 1.4E-05	N/A	

		**MSI Pathway**			

	***J*_0_**	***J*_1_**	***J*_2_**	***J*_3_**	***J*_4_**
Mutation Rate	8.3E-07	3.5E-04	1.4E-03	9.3E-03	7.7E-06
	± 1.38E-08	± 1.89E-05	± 8.57E-05	± 1.22E-03	± 1.7 9E-06
Proliferation Rate	0	0	0	2.8E-03	2.0E-02
	N/A	N/A	N/A	± 7.01E-04	± 3.31E-04
Birth Rate	0	0	0	9.6E-03	2.6E-02
	N/A	N/A	N/A	± 6.08E-04	± 2.88E-04
Growth Limiting Para.	N/A	N/A	N/A	1.6E-03	N/A
	N/A	N/A	N/A	± 3.7E-04	N/A

From these results, we have made the following observations:

(a) As shown by results in Table 2, the predicted number of cancer cases are very close to the observed cases in all age groups. This indicates that the model fits the data well and that one can safely assume that the human colon cancer can be described by a model of 2 pathways. The AIC (Akaike Information Criterion) and the BIC (Bayesian Information Criterion) from the model are 55.96 and 81.30 which are smaller than the AIC of 816.0667 and the BIC value of 827.1513 from a single pathway 4-stage model respectively (Luebeck and Moolgavkar [17]). This shows that the multiple pathway model fits better than the single pathway 4-stage model as proposed by Luebeck and Moolgavkar [17].

(b) From Table 2, it is observed that the largest number of cancer cases is in the age group between 70 and 75 years old. Comparing the values of *Q*_*i*_(*j*) between the CIN pathway (*i *= 1) and the MSI pathway (*i *= 2), it appears that the largest cancer cases is between the age group 65 and 70 years old for the CIN pathway and is between 85 and 90 years old for the MSI pathways. Presumably this might be due to the fact that the MSI pathway has one more stage than the CIN pathway.

(c) Reflecting the contribution of the APC gene on chromosomal instability, results in Table 3 showed that the mutation rates of the *I*_*r*_cells from *I*_1 _→ *I*_2 _and from *I*_2 _→ *I*_3 _had increased about 100 times and 1000 times respectively than the mutation rate from *N *→ *I*_1 _cells. Similarly, due to the contribution to genomic instability by the mis-match repair genes, the mutation rates from *J*_1 _→ *J*_2_, from *J*_2 _→ *J*_3 _and *J*_3 _→ *J*_4 _had increased about 5 * 10^2^, 0.5 * 10^4 ^and 10^4 ^times respectively than the mutation rate from *N *→ *J*_1_. Notice also from Table 3 that the mutation rates from *J*_1 _→ *J*_2 _→ *J*_3 _→ *J*_4 _are about 2 to 3 times of those from *I*_1 _→ *I*_2 _→ *I*_3_. As shown in probability plots (not shown here), these increases have speeded up the time to cancer in the MSI pathway by about 5-10 years.

(d) Results in Table 3 showed that the mutation rates from *I*_3 _→ *I*_4 _and from *J*_4 _→ *J*_5 _are of the order 10^-6 ^which were about 10^2 ^→ 10^3 ^times smaller than the mutation rates from *I*_1 _→ *I*_2 _→ *I*_3 _and from *J*_1 _→ *J*_2 _→ *J*_3 _→ *J*_4_. These results might be the consequence that we had ignored the stages of vascular carcinogenesis (i.e. angiogenesis and metastasis; see Hanahan and Weinberg [29] and Weinberg [30]) by merging these stages into the last stage. From Weinberg ([30], Chapters 13-14), notice that the angiogenesis and metastasis are also multi-stage processes.

(e) Results in Table 3 showed that the proliferation rates (birth rate - death rate) of the *I*_3 _cells and the *J*_4 _cells are of order 10^-2 ^which are much larger than the proliferation rates of the *I*_2 _cells and the *J*_3 _cells, due presumably to the effects of the silencing or inactivation of the cell cycle inhibition genes (Smad4 and TGF-*β*-RII) and the apoptosis inhibition genes (p53 and Bax). Notice from Table 3 that the estimates of the proliferation rates of the *I*_2 _and *I*_3 _cells are approximately equal to those of the *J*_3 _and *J*_4 _cells respectively. These results seemed to suggest that the genomic instabilities had little effects on cell proliferations.

## Conclusions and Discussion

Recent studies of cancer molecular biology have indicated very clearly that human colon cancer is developed through multiple pathways ([1-9]). This indicates that single pathway models are not realistic and hence may lead to incorrect prediction and confusing results. For developing efficient prevention and controlling procedures for human colon cancer and for prediction of future human colon cancer, in this paper we have developed a stochastic model and a state space model for carcinogenesis of human colon cancer involving multiple pathways with each pathway being a multi-stage model. Using this model, we have derived for the first time the probability distribution of the numbers of initiated cells and the probability distribution of time to cancer tumors. Such derivation by the traditional approach is extremely difficult and had not been attempted previously for multiple pathway models. Based on the state space model of colon cancer, we have developed a generalized Bayesian procedure to estimate the unknown parameters and to predict future cancer cases. This approach combines information from three sources: The stochastic system model via *P*{***X***, ***U***|Θ}, the prior information via *P*{Θ} and information from data via *L*{Θ|, ***X***, ***U***}. Because of additional information from the stochastic system model, our procedure is advantageous over the standard Bayesian procedure and the sampling theory procedure. Notice that there are a large number of unknown parameters in the model and only a limited amount of data are available. Without this additional information, it is then not possible to estimate all unknown parameters. Notice also that through the stochastic system model, one can incorporate biological mechanism into the model. Because the number of stages and the mutation rates of intermediate cells in different pathways are different and different drugs may affect different pathways, we believe that this is important and necessary.

We have applied these models and procedure to the NCI SEER data (upto November, 2007). Our results showed that the proposed multiple pathways model fitted better than the single pathway 4-stage model as proposed by Luebeck and Moolgavkar [17]. (The respective AIC and BIC for the multiple pathways model are 55.96 and 81.30 which are ten times smaller than those of the AIC (816.0667) and BIC (827.1513) respectively of the single pathway 4-stage model.)

In this preliminary study, we have not yet compared the multiple pathways model with the single pathway model regarding prediction of future cancer cases and evaluation of treatment protocols for human colon cancer. This will be our future research, we will not go any further here.

## Abbreviations

CIN: Chromsomal Instability; LOH: Loss of Heterozygocity; MSI: Micro-Satellite Instability; APC: Adenomatous Polyposis Coli; MMR: Mis-Match Repair; ML: Multinomial; Dev: Deviance; AIC: Akaike Information Criterion; BIC: Bayesian Information Criterion; SEER: Surveillance Epidemiology and End Results.

## Competing interests

The results of this paper have not been published in other places.

## Authors' contributions

Both authors contributed equally.

## Reviewers' comments

### Reviewer 1 (M.P. Little)

General comments

This is a generally well-written paper, describing a model very similar to that recently developed by Little et al. [12], generalising the model of Little and Wright ([11]). Arguably thislatest model should be referenced, with discussion of mathematical differences between the model outlined here and that one. There could also be discussion of the somewhat different conclusions reached in fits to more or less the same SEER colon cancer data.

**Response: **The paper by Little et al. has been added to the reference; see Little et al. [12].

Specific comments (page/line)

(1) The 6-stage model is suddenly drawn from the hat here, but is then almost immediately reduced to a 4-stage model! Why does the haplo-insufficiency of Smad4 and p53 justify combining the stages in this way? Is there evidence that Smad4 and p53 are without function at half gene dose? Also, what is the evidence for mutations being in the order given in Figure 1?

**Response: **References documenting haplo-insufficiency of p53 and smad4 have been given in the paper. The reason why we can combine the two-stages involving P53 into one-stage is based on these papers. To illustrate, let B denote the P53 gene and b the mutant of p53. Then, under haplo-insufficiency of P53, the level and effects of the P53 protein has been reduced significantly (at least 4-fold or more) so that the phenotype of genotype B/b (or B/-) is closely approximated by that of genotype b/b.

(2) How would a model incorporating epigenetic effects differ from the (DNA mutational) models already outlined? I suspect that mathematically the formalism would be exactly the same, although the implied "mutation rates" would be very much higher for epigenetic events.

**Response: **From modeling viewpoint, it is difficult to tell the difference between epigenetic changes and mutation except that the former is much more frequent and very often reversible. However, epigenetic changes can help the modeler to incorporate biological information into the biological process. Many biological papers (just during 2008, there are hundreds of biological papers published) support the epigenetic changes and the view that epigenetic changes are the driving force for cancer initiation, progression and metastasis, more important than gene mutations in cancer initiation and progression. Epigenetic changes include methylation (hypo- and hyper-methylation), micro-RNA (non-coding RNA), loss of imprinting, Histone acetylation, HDAC, tissue disorganization and gap junction disruption, etc.; epigenetic changes can also lead to gene mutations; for a brief review, see Tan and Hanin [31] (Chapter 3).

(3) This is slightly confusing. I assume that the two pathways referred to are CIN and MSI, but coming after discussion of epigenetic effects, perhaps this was meant as well or instead.

**Response: **The genetic sequence of the CIN and MSI pathways were determined by molecular biology of colon cancer and have been published in cancer journals. (There are a large number of biological papers documenting this. I have just listed a few of them in our paper. I can provide many more published papers from cancer journals if one wishes.) This sequence appears to be logical from biological mechanism. Notice that the APC-*β*- Catenin activates myc and cyclin D to push the cell into cell cycle; for the cell cycle to progress, the inhibition effects of p15, p16, p18, p19, p27 have to be abrogated through the inhibition of the TGF-*β *signaling pathway (epigenetic silencing or inactivation or mutation or deletion of smad2/smad4 (CIN pathway) or TGF-*β *Receptor II (MSI pathway).). When the number of cells has increased to certain level, then the apoptosis p53-Bax pathway is activated. For the progression of carcinogenesis, the p53 (CIN pathway) or the Bax gene (MSI pathway) have to be epigenetically silenced or inactivated or mutated or deleted. This is the reason why only the late stage involves silencing or inactivation of the gene p53. This is illustrated in our paper; see also Tan and Hanin [31] (Chapter 3, Chapter 11 and Chapter 12). There are no biological supports single-stage or two stage models. Hence it is not logical to accept single stage or two stage models for colon cancer for people who are born normal. Also, it is extremely difficult for me to accept that there are no proliferation for *I*_2 _cells as assumed in some of the cancer model papers simply because of the observation of polyps in colon which are derived from proliferation of second-stage cells and is the basis for colon cancer screening procedure "Colonoscopy" practiced by medical doctors.

(4) I assume that asymmetric mutations are assumed, in which a cell produces one normal daughter cell and one mutant daughter cell, as assumed by Little et al. [12] and Little and Wright [11] and many others, but this could perhaps be clarified. It might be useful to derive this Kolmogorov forward equation (2) in an Appendix. Clearly this forward equation (2) is in general intractable. I suspect that as in the papers of Little et al. [12] and Little and Wright [11] it is much easier to attempt to solve the Kolmogorov backward equations.

**Response: **As in Little, I assume that under genetic changes, a normal (or one *I*_*j *_cell) cell will give rise to one normal cell (or one *I*_*j *_cell) and one mutant cell (or one *I*_*j*+1_cell)(asymmetric change or mutation). This is logical because mutation or genetic changes occur during cell division.

(5) These conclusions are somewhat at odds with those of Little et al. [12] and Little and Li [32] who fitted models to very similar SEER colon cancer data and demonstrated that there was little evidence of better fit of models that allowed for genomic instability compared with those that did not. In particular the 4-stage model of Luebeck and Moolgavkar [16] provided as good fit as models that allowed for genomic instability [32], or as here multiple types of instability (CIN and MSI) [12]. The authors may care to discuss this.

**Response: **On November 13, 2009, Dr. Little had sent me his comments about our paper and a copy of his 2008 paper which I was not aware of before I wrote our paper. Dr. Little claimed that their fitting of the data could not differentiate between many different models. When I examined Dr. Little's paper, I found out that our estimation approach is very different from Dr. Little's. While Dr. Little's approach used the classical sampling theory through maximum likelihood estimates; our approach is the state space modeling and the generalized Bayesian inference incorporating information from three different sources: (1) The biological information from cancer molecular biology, (2) prior information from epigenetic and genetic mechanism, and (3) information from the likelihood function of observed data involving cancer incidence. Furthermore, because cancer incidence are derived only from people who are less than 100 years old (his/her life time), we first change the SEER data from (*n*_*j*_, *y*_*j*_) to (*m*_*j*_, *y*_*j*_), where *m*_*j*_is the number of people who can develop colon cancer during his life time (i.e., less that 100 yrars old.). Notice that *n*_*j *_is of the order 10^7 ^while *m*_*j *_is of the order 10^5^. This is described in detail in our paper in Tan and Hanin [31] (Chapter 11). Because of this and because of additional information from biological mechanism and prior distribution, we do not have the problem of identification of parameters which statisticians usually encounter in analyzing data without using information from biological mechanism. Thus, using classical approach, the parameters are not identifiable so that one has to make some assumptions such as that growth rates (birth rates) of different stage cancer initiated cells are equal which can hardly be realistic in carcinogenesis. (Biological studies by cancer biologists clearly demonstrated that the proliferation rates of cancer initiated cells with different genetic changes are very different in most of the cases.)

Finally, I want to emphazise that many models can fit the data but some fit better than others. Finally I like to emphasize what Dr. Van Ryzin had concluded 20 years ago that for cancer risk assessment, many models can fit the data but only models which are biologically supported can give correct results for cancer prediction and cancer risk assessment. It is important to list the predicted numbers along with the observed numbers in cancer modeling research. EPA has revised the guidelines to require that cancer risk assessment models should be biologically supported.

### Reviewer 2 (M. Kimmel)

The paper by Tan and Yan, proposes a new stochastic model of colon cancer progression, involving the chromosomal instability pathway and the micro-satellite instability pathway. This model not only might provide more insights into human colon cancer but also might provide useful guidance for its prevention and control and for prediction of future cancer cases. One interesting question is as to whether Tan and Yang model might help in deciding about the value of early detection of colon cancer by screening examination. Although colonoscopy is an accepted procedure, there are questions that linger concerning the impact of early detection on mortality reduction.

### Response to Reviewer 2

Thank you very much for your comments and suggestion. We are currently collecting data on screening by colonoscopy. We will apply the model to estimate cancer incidence under screening and will examine if colonoscopy will help reduce cancer incidence. We will do computer simulation to find this out. This is our next research on colon cancer.
